# Correction: Brain-Computer Interface-Based Communication in the Completely Locked-In State

**DOI:** 10.1371/journal.pbio.3000089

**Published:** 2018-12-12

**Authors:** Ujwal Chaudhary, Bin Xia, Stefano Silvoni, Leonardo G. Cohen, Niels Birbaumer

The URL where data for Patient F can be found is missing from the captions of Fig 1, Fig 2, S4 Table, S5 Table, and S2 Fig in the original article. The URL where the data can be found is: https://doi.org/10.5281/zenodo.1419151. Please see the complete captions for Fig 1, Fig 2, S4 Table, S5 Table, and S2 Fig below, along with their accompanying figure and supporting information files.

**Fig 1:**
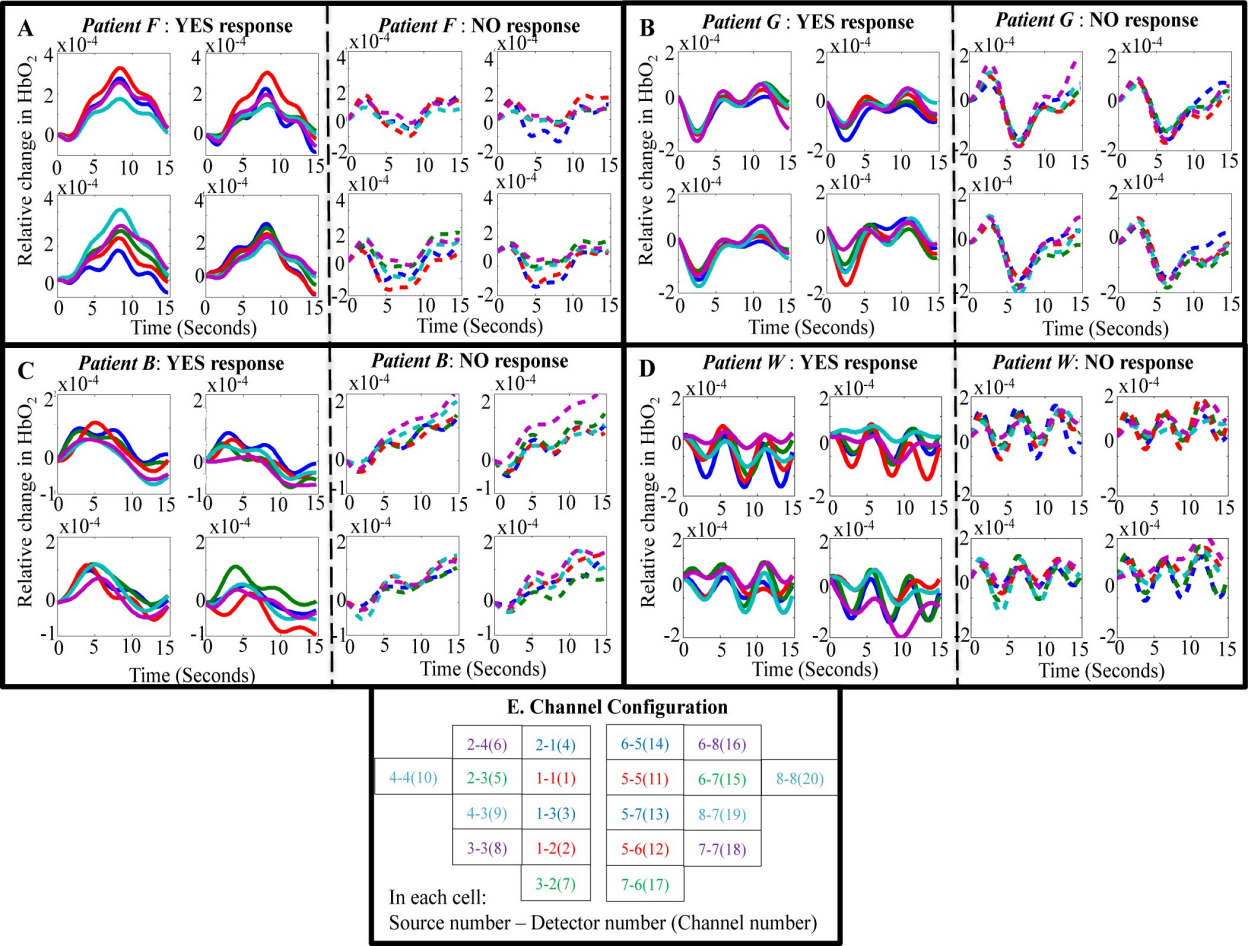
The averaged relative change in O2Hb corresponding to “yes” and “no” sentence interstimuli interval (ISI). (A) Patient F, (B) patient G, (C) patient B, and (D) patient W. (E) Channel configuration: Eight sources and eight detectors placed on the frontocentral brain region translated into 20 channels, 10 on each side of each hemisphere. For clearly displaying the relative change in O2Hb, 10 channels on each side of hemisphere were further subdivided in groups of 5 channels—i.e., 20 channels were divided into four groups, each consisting of 5 channels. In each subplot, the x-axis is time in seconds and the y-axis is relative change in O2Hb, and the five different colored lines correspond to relative change in O2Hb across 5 different channels, as depicted in the channel configuration map. Fig 1 data is located at https://doi.org/10.5281/zenodo.1419151; https://doi.org/10.5281/zenodo.192386; https://doi.org/10.5281/zenodo.192388; https://doi.org/10.5281/zenodo.192390; https://doi.org/10.5281/zenodo.192391.

**Fig 2:**
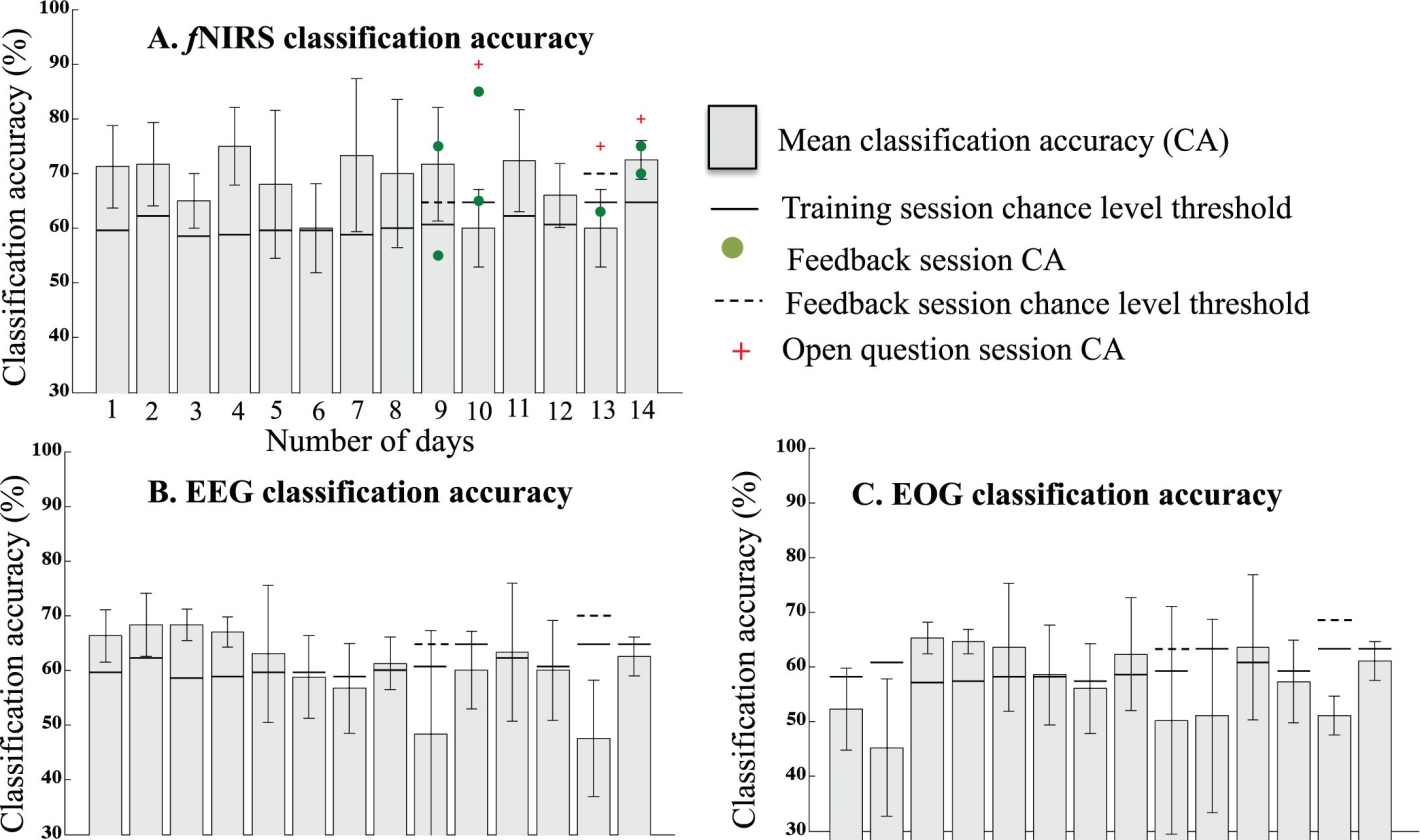
Classification accuracy of Patient F. Linear SVM CA across “training sessions—offline CA” (histogram in grey), “feedback sessions—online CA” (green dot), and “open question session—online CA” (plus sign in red), obtained using (A) relative change in O2Hb, (B) EEG, and (C) EOG data. The classification accuracy reported here is daywise, as all the “training sessions” in a day were used to calculate the average classification accuracy of all the “training sessions” in a day. In the figure panels A, B, and C, the x-axis is the number of days and the y-axis is the classification accuracy. The solid black and dotted horizontal lines represent the chance-level threshold calculated using the metric described in the BCI effectiveness metric section for “training sessions” and “feedback sessions,” respectively. Since the feedback during the feedback and open question sessions was provided using the O2Hb, the online CA of the feedback and open question sessions is reported only for the fNIRS data. Fig 2 data is located at https://doi.org/10.5281/zenodo.1419151; https://doi.org/10.5281/zenodo.191884.

## Supporting information

S4 Table Patient F. Contingency table formed using the average of all the training sessions. S4 Table data is located at https://doi.org/10.5281/zenodo.1419151; https://doi.org/10.5281/zenodo.192398.
(XLSX)

S5 Table Patient F. Contingency table formed using the average of all the feedback sessions. S5 Table data is located at https://doi.org/10.5281/zenodo.1419151; https://doi.org/10.5281/zenodo.192400.
(XLSX)

S2 Fig Patient F. Receiver operating characteristic (ROC) curve of the binary support vector machine (SVM) classifier. (A) Training and (B) feedback sessions. Each circle in the ROC curve space represents false positive rate (FPR) versus true positive rate (TPR) for each session. Sessions with the same coordinate points in the ROC space are represented by concentric circles. The red star along with the coordinate points in the ROC space represent FPR versus TPR of all the sessions combined. In the figure panels A and B, the x-axis is the FPR and the y-axis is TPR. The thick diagonal line dividing the ROC space represents chance level. Points above the diagonal represent good classification results (better than random); points below the line represent poor classification results (worse than random). S2 Fig data is located at https://doi.org/10.5281/zenodo.1419151; https://doi.org/10.5281/zenodo.192398; https://doi.org/10.5281/zenodo.192400.
(JPG)
