# Supplementary figures and images for: Correction: Brain-Computer Interface-Based Communication in the Completely Locked-In State
Source: PLoS Biol. 2018 Dec 12;16(12):e3000089. doi: 10.1371/journal.pbio.3000089 (PMC6291112; doi:10.1371/journal.pbio.3000089)

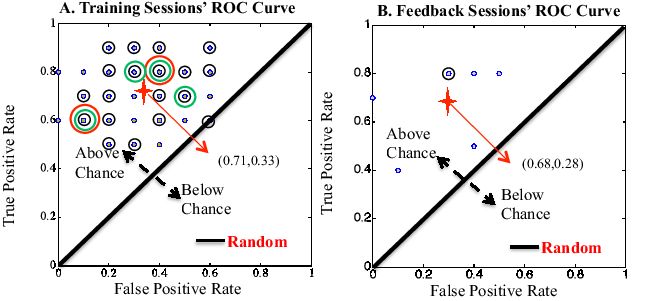

Supplement: S2 Fig — Receiver operating characteristic (ROC) curve of the binary support vector machine (SVM) classifier. (A) Training and (B) feedback sessions. Each circle in the ROC curve space represents false positive rate (FPR) versus true positive rate (TPR) for each session. Sessions with the same coordinate points in the ROC space are represented by concentric circles. The red star along with the coordinate points in the ROC space represent FPR versus TPR of all the sessions combined. In the figure panels A and B, the x-axis is the FPR and the y-axis is TPR. The thick diagonal line dividing the ROC space represents chance level. Points above the diagonal represent good classification results (better than random); points below the line represent poor classification results (worse than random). S2 Fig data is located at https://doi.org/10.5281/zenodo.1419151; https://doi.org/10.5281/zenodo.192398; https://doi.org/10.5281/zenodo.192400. (JPG) [file pbio.3000089.s003.jpg]
